# Gut-derived bacterial toxins impair memory CD4^+^ T cell mitochondrial function in HIV-1 infection

**DOI:** 10.1172/JCI149571

**Published:** 2022-05-02

**Authors:** Brian Ferrari, Amanda Cabral Da Silva, Ken H. Liu, Evgeniya V. Saidakova, Larisa B. Korolevskaya, Konstantin V. Shmagel, Carey Shive, Gabriela Pacheco Sanchez, Mauricio Retuerto, Ashish Arunkumar Sharma, Khader Ghneim, Laura Noel-Romas, Benigno Rodriguez, Mahmoud A. Ghannoum, Peter P. Hunt, Steven G. Deeks, Adam D. Burgener, Dean P. Jones, Mirela A. Dobre, Vincent C. Marconi, Rafick-Pierre Sekaly, Souheil-Antoine Younes

**Affiliations:** 1 Department of Medicine, Division of Infectious Diseases and HIV Medicine, Center for AIDS Research, Case Western Reserve University/University Hospitals Cleveland Medical Center, Cleveland, Ohio, USA.; 2 Department of Pathology, Pathology Advanced Translational Research (PATRU), School of Medicine and; 3 Clinical Biomarkers Laboratory, Department of Medicine, Emory University, Atlanta, Georgia, USA.; 4 Institute of Ecology and Genetics of Microorganisms, Perm Federal Research Center Ural Branch Russian Academy of Sciences, Perm, Russia.; 5 Department of Microbiology and Immunology, Perm State University, Perm, Russia.; 6 Cleveland VA Medical Center, Cleveland, Ohio, USA.; 7 Integrated Microbiome Core, Department of Dermatology, Case Western Reserve University/University Hospitals Case Medical Center, Cleveland, Ohio, USA.; 8 Center for Global Health and Diseases, Case Western Reserve University, Cleveland, Ohio, USA.; 9 Department of Medicine, UCSF, San Francisco, California, USA.; 10 Department of Obstetrics & Gynecology, University of Manitoba, Winnipeg, Manitoba, Canada.; 11 Department of Medicine (Nephrology), Case Western Reserve University/University Hospitals Cleveland Medical Center, Cleveland, Ohio, USA.; 12 Division of Infectious Diseases, Department of Global Health, and Department of Global Health, Rollins School of Public Health, Atlanta, Georgia, USA.

**Keywords:** AIDS/HIV, Infectious disease, Apoptosis, Mitochondria, T cell development

## Abstract

People living with HIV (PLWH) who are immune nonresponders (INRs) are at greater risk of comorbidity and mortality than are immune responders (IRs) who restore their CD4^+^ T cell count after antiretroviral therapy (ART). INRs have low CD4^+^ T cell counts (<350 c/**μ**L), heightened systemic inflammation, and increased CD4^+^ T cell cycling (Ki67^+^). Here, we report the findings that memory CD4^+^ T cells and plasma samples of INRs from several cohorts are enriched in gut-derived bacterial solutes *p*-cresol sulfate (PCS) and indoxyl sulfate (IS) that both negatively correlated with CD4^+^ T cell counts. In vitro PCS or IS blocked CD4^+^ T cell proliferation, induced apoptosis, and diminished the expression of mitochondrial proteins. Electron microscopy imaging revealed perturbations of mitochondrial networks similar to those found in INRs following incubation of healthy memory CD4^+^ T cells with PCS. Using bacterial *16S* rDNA, INR stool samples were found enriched in proteolytic bacterial genera that metabolize tyrosine and phenylalanine to produce PCS. We propose that toxic solutes from the gut bacterial flora may impair CD4^+^ T cell recovery during ART and may contribute to CD4^+^ T cell lymphopenia characteristic of INRs.

## Graphical abstract

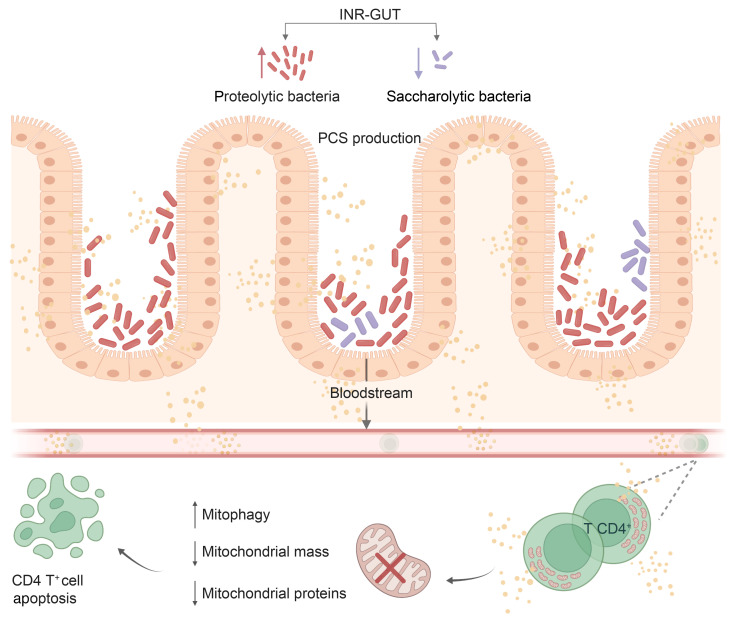

## Introduction

Despite effective control of HIV-1 replication with antiretroviral therapy (ART), a significant proportion of treated persons fails to increase CD4^+^ T cell counts to levels observed in individuals without HIV-1 infection (1, 2). These immune nonresponders (INRs) remain at greater risk for morbidity and mortality than do immune responders (IRs) in whom CD4^+^ T cell counts are restored following ART (3, 4). INRs have a profound increase in the proportions of effector memory CD4^+^ T cells in circulation (1, 5, 6) and their CD4^+^ T cells have a poor in vitro response to IL-7 (7–10). Despite low CD4^+^ T cell numbers and poor IL-7 responsiveness, an increased frequency of HLA-DR^+^/CD38^+^ and of cycling/activated (Ki67^+^) CD4^+^ T cells is characteristic of INRs (1, 2, 11). Nadir CD4^+^ T cell counts (12, 13), longer duration of HIV-1 infection (14, 15), microbial translocation and inflammation (1, 16), HIV-1 DNA persistence as well as PD-1 and TIGIT expression in long-lived CD4^+^ T cells (17), and poor responses to IL-7 and IL-15 (7–10, 17) have all been associated with failure to restore CD4^+^ T cell numbers in INR patients. As reported by our group (11), the INR phenotype is characterized by memory CD4^+^ T cell mitochondrial dysfunction regardless of nadir CD4^+^ T cell counts, late ART initiation, and patient age, and in vitro spontaneous death of cycling (Ki67^+^) memory CD4^+^ T cells occurs (11). The cause of this mitochondrial dysfunction is currently unknown.

Gut microbiota is a major player in shaping the immune system (18). People living with HIV (PLWH) have elevated levels of bacterial translocation (19–22) and show evidence of gut bacterial dysbiosis (18, 23–27). Generally, HIV-1 infection induces alterations in the gut microbiota, characterized by overall decrease in bacterial diversity (23). For instance, the gut microbial composition of PLWH not receiving ART was significantly different from HIV-1–negative persons and some bacterial genera were depleted in ART-naive HIV-1–positive patients, while others were enriched (28). Markers of bacterial translocation are elevated in the plasma of INRs versus IRs (1, 29), and it was reported that gut microbial diversity is in dysbiosis and rich in *Fusobacterium* (30) or *Faecalibacterium prausnitzii*, unclassified *Subdoligranulum spp*., and *Coprococcus* (31). All these studies, however, are based on phyla or genera abundance and do not provide a direct mechanism of how the bacterial dysbiosis in PLWH affects CD4^+^ T cell homeostasis in INRs. It is widely accepted that bacterial translocation from the gut to the bloodstream plays a major role in the heightened inflammation detected in PLWH (32); recently, *Serratia* genera DNA detection in the plasma was associated with levels of inflammatory mediators such as IL-1, IL-6, and IL-8, thus validating the role of gut bacterial translocation in the heightened inflammation reported in HIV-1 disease and specifically in INRs (32).

Gut bacteria–derived solutes (GBDSs) such as *p*-cresol sulfate (PCS) and indoxyl sulfate (IS) are the most studied so-called “uremic toxins” that accumulate in the plasma of patients with chronic kidney disease (CKD) and associate with cardiovascular and kidney diseases (33–35). Four GDBSs are generated by gut microbiota and have been extensively studied in combination with adverse clinical outcomes: (a) PCS is generated from tyrosine and phenylalanine as *p*-cresol by the gut microbiota and then acquires the sulfate group in the colon and the liver; (b) IS is generated from tryptophan by the gut bacterial tryptophanase enzyme, which transforms tryptophan to indole and then acquires the sulfate group in the colon and liver as well; (c) phenylactylglutamine (PAG) is generated by the gut microbiota from phenylalanine; and (d) trimethylamine *N*-oxide (TMAO) is generated by the gut microbiota from dietary amines, choline, betaine, and carnitine (36). Few studies have addressed the implication of these GDBSs in the modulation of immune responses (37) or whether GDBSs have a role in CD4^+^ T cell depletion in INRs.

In this study, we report what we believe are novel findings that the GDBSs, PCS and IS, impair mitochondrial fitness of CD4^+^ T cells and may thereby contribute to the CD4^+^ T cell lymphopenia characteristic of INRs. We provide evidence that stool samples of INRs are enriched in bacterial genera capable of PCS production.

## Results

### Mitochondrial defects in INRs modulate all metabolic pathways.

We previously reported mitochondrial dysfunction in memory CD4^+^ T cells of INRs as detected by transcriptomic and flow cytometric analysis (11). To further investigate the mitochondrial dysfunction, we sorted memory CD45RO^+^CD4^+^ T cells from 14 INRs, 15 IRs, and 7 healthy controls (HCs) from the Russian Perm cohort (Supplemental Table 1; supplemental material available online with this article; https://doi.org/10.1172/JCI149571DS1) and monitored the levels of 165 metabolites reflective of distinct metabolic pathways using ultrahigh-performance liquid chromatography–tandem mass spectroscopy (UPLC-MS/MS) assays. Levels of these metabolites were normalized to cell lysate protein concentrations and compared among the groups. Out of the 165 metabolites detected, 127 were statistically significantly diminished or enriched among the groups after *P*-value correction with the Benjamini-Hochberg test.

Elevated levels of TCA intermediates can reflect changes in carbon inflow or outflow. Carbon can flow into the TCA cycle from several sources, including carbohydrates and lipids (via conversion of acetyl-CoA to citrate), glutamine (entering as α-ketoglutarate), and branched-chain amino acids (entering as acetyl-CoA and succinyl-CoA) (38). Our data show that citrate and aconitate were elevated in the INR samples compared with HCs and IRs (Supplemental Figure 1A). While this could indicate increased acetyl-CoA input, there was no evidence of elevated glycolysis. In fact, 3-phosphoglycerate, phosphoenolpyruvate (PEP), and glycerate, all intermediates in the glycolysis pathway, show decreased levels in the INR samples, consistent with a decrease in glycolytic activity (Supplemental Figure 1B). Nicotinamide (the precursor of NAD) was significantly diminished in INR samples, indicative of a dysfunctional TCA cycle. Lastly, the later TCA cycle intermediates fumarate and malate were found to be significantly lower in the INR samples. This would also suggest an overall decrease in TCA cycle activity, possibly leading to accumulation of the earlier intermediates citrate and aconitate. Overall, these data validate our previous transcriptomic and flow cytometric observations that oxidative phosphorylation is diminished in INR memory CD4^+^ T cells (11). Our data also show that amino acid metabolism (Supplemental Figure 2), lipid (Supplemental Figure 3), phospholipid (Supplemental Figure 4), sphingomyelin (Supplemental Figure 5), and nucleotide (Supplemental Figure 6) pathways were all altered in the INR samples, possibly due to the mitochondrial dysfunction characteristic of the INR group. In fact, the mitochondrial network is implicated in all major metabolic pathways (glycolysis, pentose pathway, amino acid metabolism, lipids synthesis, and fatty acid oxidation) by providing essential cofactors (39).

### PCS and IS are enriched in INR patients.

**Figure 1:**
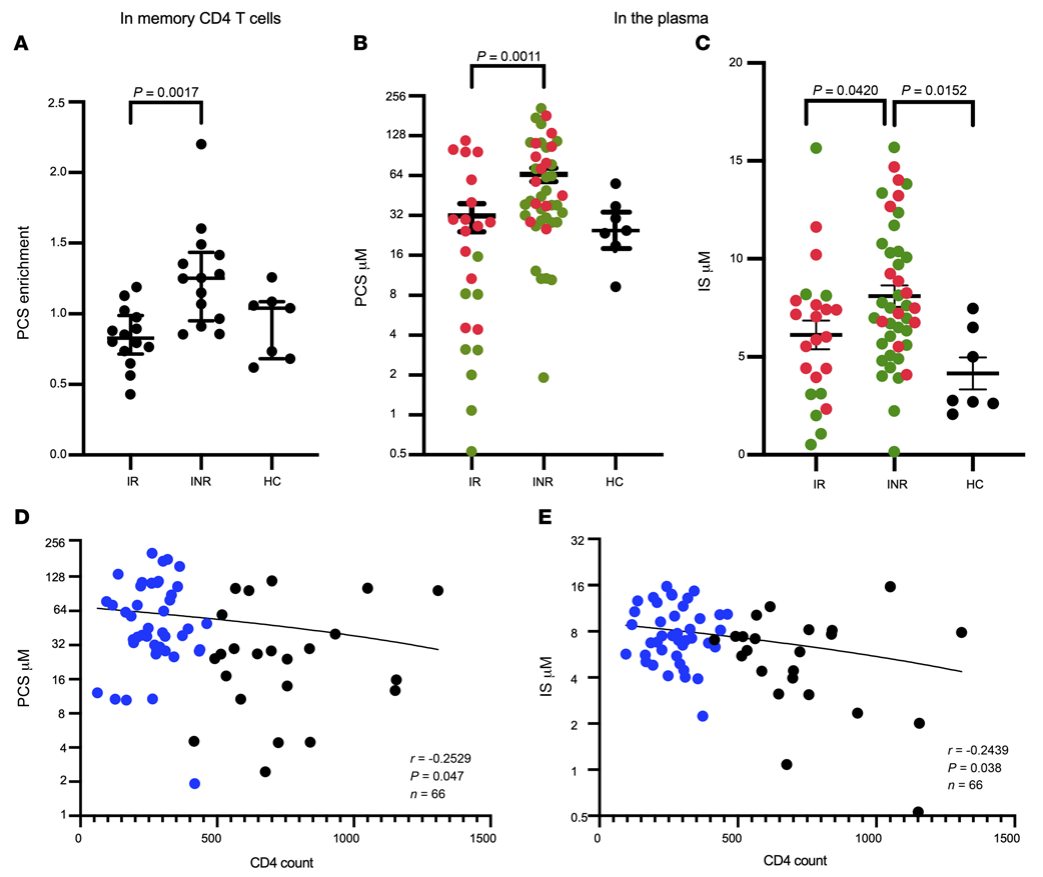
Enrichment of PCS and IS in INR samples. Untargeted metabolic UPLC-MS/MS measurements showing levels of PCS (**A**) in 1 × 10^6^ to 2 × 10^6^ sorted memory CD4^+^ T cells of IRs (*n =* 15), INRs (*n =* 14), and HCs (*n =* 7). Concentration of PCS (**B**) and IS (**C**) in the plasma of the IR and INR groups in the CLIF (red dots) and SCOPE (green dots) cohorts. Median and interquartile range (solid lines) are displayed in the dot plots. *P* values were generated by Kruskal-Wallis with Dunn’s multiple-comparison test. Spearman’s correlation of plasma concentrations of PCS (**D**) and IS (**E**) with CD4^+^ T cell count. Blue dots are INR patient data.

The metabolic analysis performed here allowed us to detect PCS significant enrichment (*P* = 0.0017) in memory CD4^+^ T cells of INRs (Figure 1A). We also found enriched concentrations of PCS (Figure 1B) and IS (Figure 1C) in the INR plasma of 2 independent cohorts from different clinical sites: the SCOPE (UCSF) and CLIF (CWRU/UH Cleveland) cohorts (Supplemental Tables 2 and 3). PAG was not detected and TMAO levels were not statistically significantly different when compared among IRs and INRs (Supplemental Figure 3). Importantly, CD4^+^ T cell counts were negatively associated with PCS (Figure 1D) and IS (Figure 1E) plasma concentrations. These data suggest that PCS and IS may modulate CD4^+^ T cell homeostasis by impeding CD4^+^ T cell survival.

The average serum/plasma concentrations of PCS reported in HCs varies between 14.9 μM and 35.1 μM, whereas IS concentrations are 5- to 8-fold lower as determined by UPLC and UPLC-MS/MS, respectively (40, 41). Our data are in agreement with the reported studies, as median PCS concentrations were 31.69 ± 7.63 μM in IRs, 65.39 ± 7.66 μM in INRs, and 28.53 ± 5.61 μM in HCs (Figure 1B), whereas IS concentration median values were 6.12 ± 0.73 μM in IRs, 8.10 ± 0.54 μM in INRs, and 4.16 ± 0.81 μM in HCs (Figure 1C). These data demonstrate that levels of PCS were significantly 2-fold higher in the plasma of INRs compared with IRs, whereas IS concentration values were significantly 1.3-fold higher in INRs versus IRs.

### PCS and IS plasma concentrations correlate with markers of inflammation.

**Figure 2:**
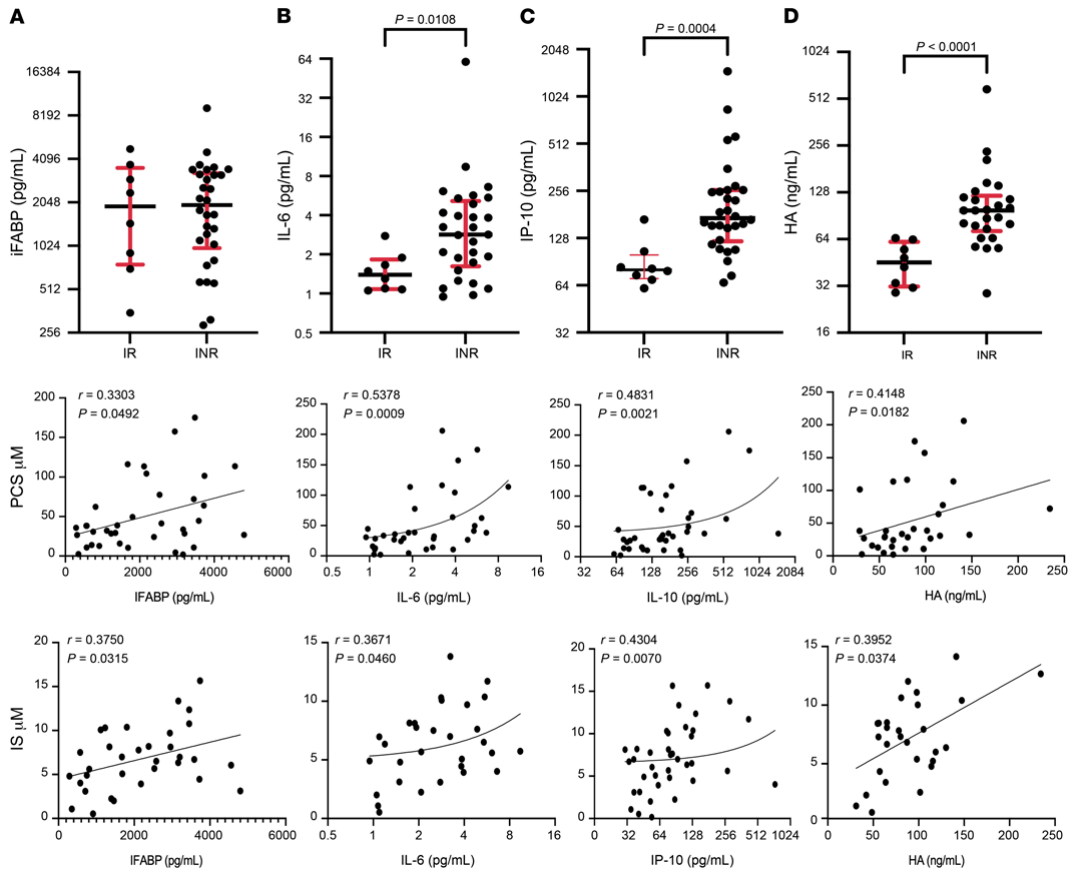
Correlation of PCS and IS plasma concentration with markers of inflammation. (**A**) Plasma concentrations of IFABP in IRs and INRs and Spearman’s correlation between PCS (middle panels)/IS (lower panels) and IL-6 (**B**), IP-10 (**C**), and hyaluronic acid (HA) (**D**). Median and interquartile range (in red) are shown.

We next examined the correlation of plasma levels of PCS and IS with soluble inflammatory mediators (IL-6, IFN-γ–induced protein 10 [IP-10], hyaluronic acid [HA]), the intestinal injury marker intestinal fatty acid–binding protein (IFABP), and bacterial translocation makers (LPS and soluble CD14 [sCD14]). As predicted and shown previously, IL-6, IP-10, and sCD14 (1, 42) were significantly elevated in plasma of INRs compared with IRs, yet levels of PCS and IS were not significantly correlated with plasma levels of sCD14 or LPS (Supplemental Figure 7, A and B) but rather with levels of IFABP (Figure 2A), IL-6 (Figure 2B), IP-10 (Figure 2C), and HA (Figure 2D). These data suggest that PCS and IS levels correlate with inflammatory mediators rather than markers of bacterial translocation. The positive correlation between IFABP plasma levels and PCS/IS plasma concentrations suggest that gut epithelial cell damage may facilitate PCS and IS passage to the blood stream.

### PCS and IS block CD4^+^ T cell proliferation, induce apoptosis, and diminish mitochondrial protein expression.

**Figure 3:**
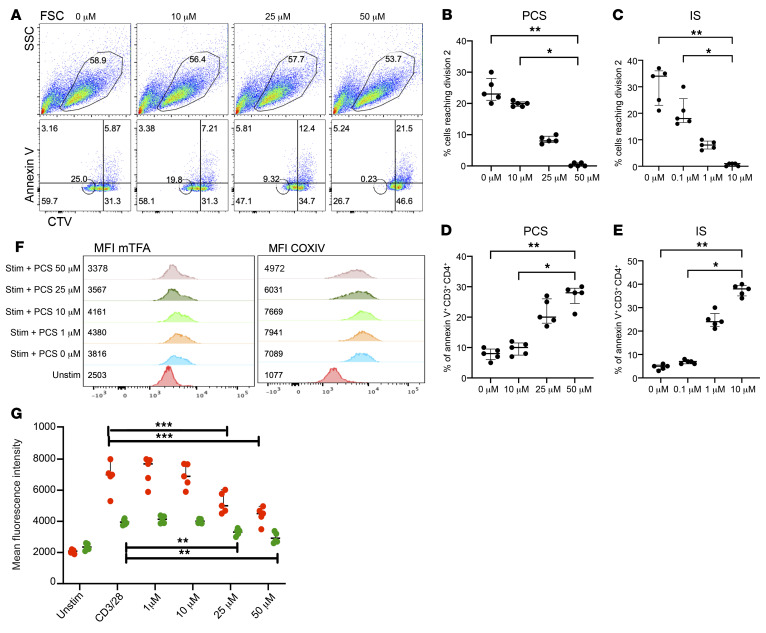
PCS and IS block CD4^+^ T cell proliferation, increase apoptosis levels, and diminish mitochondrial protein expression in a dose-dependent manner. (**A**) Representative CTV dye dilution proliferation blockade upon treating PBMCs of a healthy donor with gradient concentrations of PCS. CTV-labeled PBMCs were stimulated with anti-CD3/anti-CD28 in media with 2% FBS followed by treatment with increasing concentrations of PCS. CTV dye dilution was evaluated at 72 hours. Apoptosis levels were measured by annexin V binding. Cumulative data (*n =* 5) on the effect of PCS (**B**) and IS (**C**) on CD4^+^ T cell proliferation as monitored by CTV dye dilution. Cumulative data (*n =* 5) for the effect of PCS (**D**) and IS (**E**) on the levels of annexin V binding. (**F**) Effect of PCS on protein expression of mTFA and COXIV implicated in mitochondrial function. (**G**) The cumulative MFI (*n =* 5) for mTFA (green dots) and COXIV (red dots). Median and SD are displayed. *P < 0.05; **P < 0.01; ***P < 0.001 by Kruskal-Wallis with Dunn’s multiple-comparison test.

To analyze the effect of PCS and IS on CD4^+^ T cells, we stimulated PBMCs from 5 healthy donors with anti-CD3/anti-CD28 antibodies followed by treatment with physiological-range gradients of PCS (10–50 μM) and IS (0.1–10 μM) in 2% FBS–containing media, as we found that excess FBS quenches PCS and IS effects in vitro (data not shown). Cells were incubated for 72 hours followed by flow cytometric analysis. As depicted in Figure 3A, cell integrity assessed by side and forward scatter (SSC/FSC) was not affected even at the highest concentration of PCS (50 μM). In contrast, annexin V binding on CD4^+^ T cells increased proportionally with higher concentrations of PCS/IS (Figure 3, A, D, and E). To monitor the impact of PCS/IS on CD4^+^ T cell proliferation, Cell Trace Violet (CTV) dye dilution was evaluated following treatment with increasing concentrations of PCS and IS. As shown in Figure 3, A–C, CD4^+^ T cells were prevented from reaching a second round of cell division after PCS and IS exposure in vitro. To assess the impact of PCS and IS treatment on CD4^+^ T cell mitochondria, we evaluated the mean fluorescence intensities (MFIs) of the terminal enzyme of the mitochondrial respiratory chain, cytochrome *c* oxidase subunit 4 (COXIV), and the mitochondrial transcriptional factor A (mTFA) implicated in mitochondrial DNA replication. As shown in Figure 3, F and G, there was a significant dose-dependent reduction in mitochondrial protein expression upon treatment with PCS or IS. Altogether, these data suggest that PCS and IS block CD4^+^ T cell proliferation, diminish mitochondrial protein expression (COXIV and mTFA), and induce CD4^+^ T cell apoptosis.

**Figure 4:**
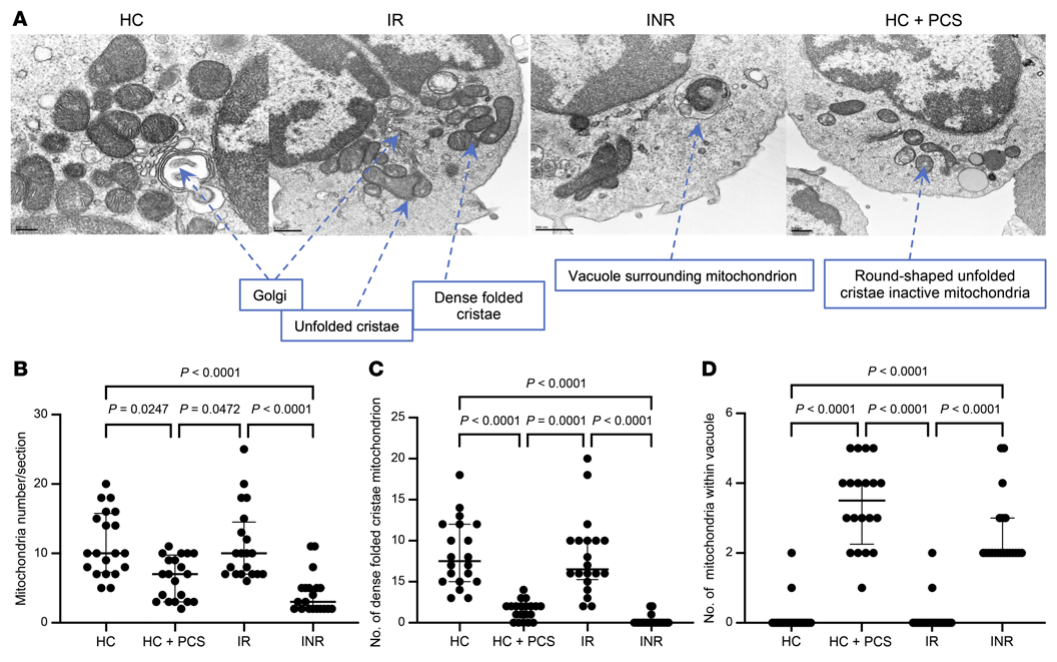
Treatment of memory CD4^+^ T cells with PCS reduces mitochondrial fitness. (**A**) Representative electron microscopy imaging (EMI) from 1 EM section of sorted memory CD4^+^ T cells from HC, HC+PCS, IR, and INR samples. Scale bars: 200 nm (left image) and 500 nm (right 3 images). (**B**) Mitochondrial numbers per EMI section. (**C**) Numbers of mitochondria with dense folded cristae. (**D**) Numbers of mitochondria with vacuoles. Twenty EMI sections from 2 independent experiments were analyzed. *P* values generated by Kruskal-Wallis with Dunn’s multiple-comparison test. Median and interquartile range are shown.

We then sorted memory CD4^+^ T cells from 2 HCs, 2 IRs, and 2 INRs and visualized the mitochondrial structure by electron microscopy imaging (EMI) to monitor the impact of PCS on mitochondrial fitness. Specifically, we monitored the shape of the mitochondria as well as the cristae density, as these features are reflective of mitochondrial function (43). As shown in Figure 4A, mitochondrial shape and cristae density reflective of active mitochondria were detected in the HC and IR EMI sections. In contrast, the INR samples displayed elongated and vacuole-surrounded mitochondria, a process known as mitophagy, which is a marker of cellular oxidative stress (44). When mitochondria were counted in each EMI section, the INR and the HC samples treated with PCS displayed the lowest mitochondrial numbers compared with findings among IR or HC samples (Figure 4B). Of note is the fact that the incubation of memory CD4^+^ T cells from an HC with 25 μM PCS for 72 hours induced mitochondrial structure perturbations similar to those detected in INR samples with rested cristae, elongated mitochondria, and low mitochondrial numbers per EMI section (Figure 4, A and B). In addition, the numbers of dense, folded cristae (Figure 4C) were significantly diminished in the INR and HC+PCS EMI sections. Lastly, the numbers of mitochondria surrounded by vacuoles (indicative of mitophagy) were significantly elevated in INR and HC+PCS sections (Figure 4D).

Altogether, these data indicate that genes (11) and proteins implicated in mitochondrial function are diminished in memory CD4^+^ T cells of INRs and the treatment of HC cells with PCS or IS diminishes mitochondrial fitness.

### The gut flora is enriched in bacterial genera able to produce PCS in INRs.

**Figure 5:**
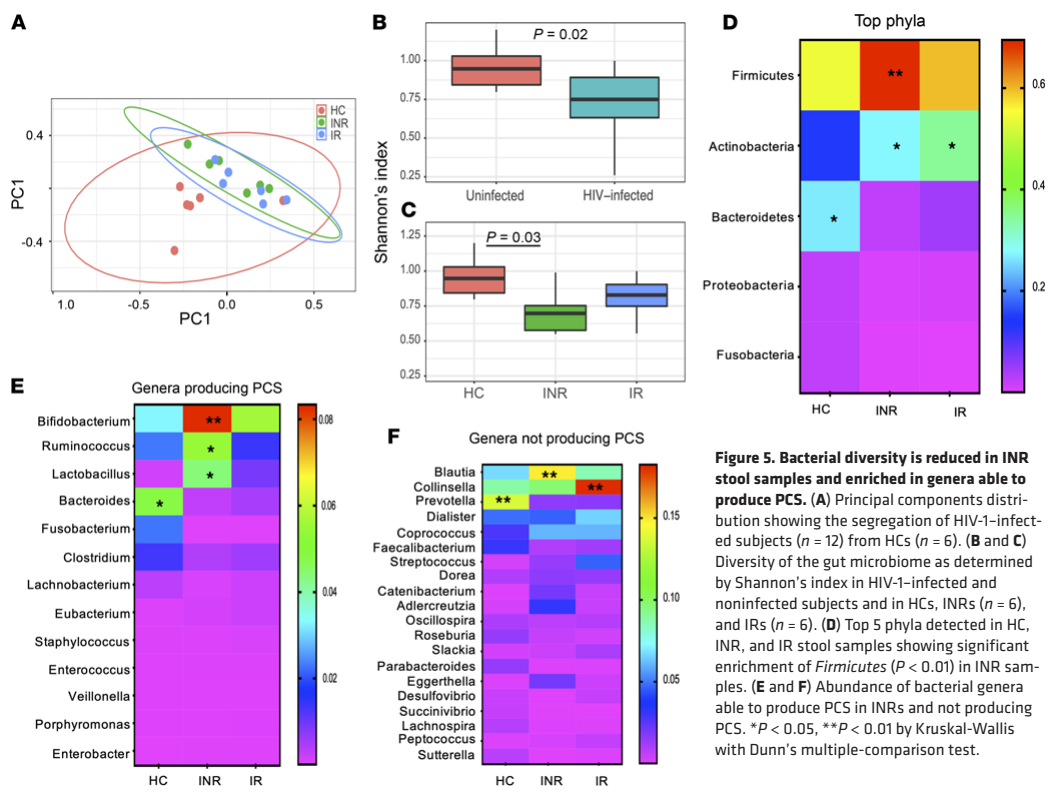
Bacterial diversity is reduced in INR stool samples and enriched in genera able to produce PCS. (**A**) Principal components distribution showing the segregation of HIV-1–infected subjects (*n =* 12) from HCs (*n =* 6). (**B** and **C**) Diversity of the gut microbiome as determined by Shannon’s index in HIV-1–infected and noninfected subjects and in HCs, INRs (*n =* 6), and IRs (*n =* 6). (**D**) Top 5 phyla detected in HC, INR, and IR stool samples showing significant enrichment of *Firmicutes* (*P <* 0.01) in INR samples. (**E** and **F**) Abundance of bacterial genera able to produce PCS in INRs and not producing PCS. *P < 0.05, **P < 0.01 by Kruskal-Wallis with Dunn’s multiple-comparison test.

PCS and IS are normally cleared by functional kidneys and both accumulate in patients with CKD (36, 45). Our multi-INR cohorts showed no signs of kidney disease as indicated by the glomerular filtration rate (eGFR) test (Supplemental Figure 7C). Thus, we hypothesized that INR stool would be enriched in bacteria capable of producing PCS, which would result in PCS accumulation in the INR plasma samples. As recognized so far, intestinal bacteria capable of generating *p*-cresol, the precursor of PCS, mainly belong to the 13 genera *Bacteroidaceae*, *Bifidobacteriaceae*, *Clostridiaceae*, *Enterobacteriaceae*, *Enterococcaceae*, *Eubacteriaceae*, *Fusobacteriaceae*, *Lachnospiraceae*, *Lactobacillaceae*, *Porphyromonadaceae*, *Staphylococcaceae*, *Ruminococcaceae*, and *Veillonellaceae* (34). We analyzed unpublished data generated by our group on previously recruited 6 HCs, 6 INRs (CD4^+^ T cells <350/μL), and 6 IRs (CD4^+^ T cells >500/μL) from whom fecal samples were collected (Supplemental Table 4). Fecal DNA was extracted and sequenced by Ion-Torrent using primers specific for bacterial *16S* and we analyzed the data for the presence of genera able to produce PCS. As shown in Figure 5A, principal components distribution segregated HIV-1–infected subjects from HCs, suggesting that the gut microbiota is distinctive between HCs and HIV-1–infected populations. The diversity of the commensal microbiota as determined by Shannon’s index was diminished in HIV-1–infected persons (Figure 5B) and in INR samples (Figure 5C). The phyla enrichment analysis revealed that the INR samples were significantly enriched in *Firmicutes* and *Actinobacteria* (Figure 5D), whereas the phylum *Bacteroidetes* was misrepresented in both IR and INR samples. These data are in accordance with the observation that *Bacteroidetes* is decreased in HIV-1–infected subjects’ gut flora (16). As shown in Figure 5E, the INR samples were enriched in the genera *Bifidobacterium* (phylum *Actinobacteria*), *Ruminococcus* (phylum *Firmicutes*), and *Lactobacillus* (*Firmicutes*), all reported to produce PCS. INR samples were also enriched in the genus *Blautia* (*Firmicutes*), suggestive of INR gut bacterial dysbiosis, yet *Blautia* is not listed among the genera able to produce PCS. Taken together, these data suggest that the enrichment of PCS in memory CD4^+^ T cells or in the plasma of INR patients could be a consequence of enrichment of gut bacterial flora having the capacity for PCS production.

## Discussion

In this report, we provide evidence that GDBSs are enriched in memory CD4^+^ T cells (PCS) and in the plasma of INR (PCS and IS), possibly impeding CD4^+^ T cell recovery. PCS is the most studied toxin and was reported to have a pleiotropic effect on multiple cell types in vitro (37). PCS is proinflammatory and alters endothelial cell function (46). PCS stimulates tumor necrosis factor-α, IL-6, and IL-1β mRNA expression in THP-1 cells as quantified by RT-PCR (47). PCS impairs mitochondrial dynamics and function of renal tubular cells (48) and increases the percentage of leucocytes displaying oxidative burst activity at baseline (49). Our in vitro data agreed with and extended these findings, as we showed that the treatment of HC PBMCs with PCS or IS blocks cell proliferation, induces apoptosis, diminishes mitochondrial protein expression in CD4^+^ T cells, and reduces mitochondrial fitness. Altogether, these data demonstrate that PCS and IS impede CD4^+^ T cell mitochondrial function and block CD4^+^ T cell proliferation in vitro. Whether PCS or IS blocks mitochondrial function and T cell proliferation in vivo requires further investigation, as few reports have analyzed the in vivo impact of PCS or IS on immune cells. For instance, in an in vivo study in which mice were fed a tyrosine-rich diet, PCS accumulated in the blood and this was associated with decreased Th1 CD4^+^ and CD8^+^ T cell responses; it was also shown that high concentrations of PCS suppressed CD4^+^ and CD8^+^ T cell IFN-γ production (50). The same group in a separate study showed that in a CKD mouse model high levels of PCS reduced the numbers of peripheral B and NK cells (51). In addition, PCS induces macrophage activation but interferes with antigen processing, leading to a failure in the adaptive immune response (52, 53). Thus, PCS indeed has a pleiotropic effect; however, PCS uses the cell surface organic anion transporter 1 (OAT-1) and OAT-3 receptors to enter cells (54). It has been demonstrated (55, 56) and validated by our group (data not shown) that OAT-1 and OAT-3 receptors are not expressed on PBMCs in vitro and as shown in Supplemental Figure 8, PCS impedes all immune cells. In addition, PCS was found enriched in memory CD4^+^ T cell lysates of INRs compared with findings among IRs or HCs (Figure 1A). Thus, it is highly plausible that PCS uses an unknown receptor(s) on PBMCs and CD4^+^ T cells. The identification of PCS receptor(s) requires further efforts and could lead to receptor-binding interference to block PCS entry into CD4^+^ T cells.

PCS and IS are exclusive products of the gut bacterial flora from tyrosine, phenylalanine (PCS), and tryptophan (IS) metabolism by commensal bacteria, as germ-free mice show no evidence of PCS or IS in the blood (34). We have provided evidence that plasma levels of PCS and IS correlate with levels of inflammatory (e.g., IL-6, IP-10) rather than bacterial translocation markers (sCD14, LPS) (Figure 2), suggesting that toxins produced by the commensal bacteria (e.g., PCS and IS) participate in the heightened inflammation detected in INRs independently of bacterial translocation. It is also possible that the enrichment of PCS or IS in INRs is the consequence rather than the cause of inflammation. As we show in Figures 3 and 4, however, PCS and IS impeded mitochondrial function, which is often associated with heightened inflammation by the generation of reactive oxygen species (ROS) production, mitophagy (Figure 4D), and cytoplasmic mitochondrial DNA release, which altogether are major sources of oxidative stress and cell death (57–61). Interestingly, members of our consortium have recently found elevated levels of *Serratia* genera by DNA deep sequencing (PathSeq) in INR plasma, suggesting that the translocation of *Serratia* to the bloodstream is accompanied by elevated levels of inflammatory cytokines (IL-1, IL-6, and IL-8) possibly as a consequence of monocyte and macrophage activation by *Serratia* products (32). We did not find *Serratia* in our study in the stool of INRs and this may be due to the different techniques used to identify *Serratia* (plasma PathSeq vs. stool *16S*); nevertheless, according to our UniProt search (www.uniprot.org), *Serratia* possesses all required enzymes to produce PCS from tyrosine (tyrosine transaminase and 4-hydroxyphenylacetate decarboxylase) and IS from tryptophan (tryptophanase). Thus, it is likely that bacterial translocation and toxin production may synergistically contribute to the heightened inflammation detected in INRs and CD4^+^ T cell depletion.

PCS and IS are cleared by the kidneys in healthy individuals and accumulate in patients with CKD (62). Moreover, patients with kidney failure have the lowest CD4^+^ T cell counts and positive correlations were established between eGFR and CD4^+^ T cell counts in HIV-1–infected patients (63–65). The INR cohorts studied here showed no sign of kidney dysfunction as evaluated by eGFR measurement (Supplemental Figure 7C). Creatinine-based eGFR, however, is a notoriously weak estimator of the early tubular injury that may occur in HIV-1 infection (66), and further assessment of kidney function is needed to rule out kidney dysfunction in our INR cohorts. Nevertheless, accumulation of PCS in blood without evidence of kidney failure has been evident in germ-free mice fed with tyrosine (50, 51), suggesting that diet may modulate levels of circulating PCS. In fact, healthy vegetarians have 62% lower urinary PCS compared with individuals consuming an unrestricted diet (67) and this is due to enrichment of saccharolytic over proteolytic bacteria, which implies less PCS production by the commensal bacterial flora. In this report, we have provided evidence that stool samples from INRs are enriched in bacterial genera able to produce PCS (Figure 5). Although we have no data reporting the levels of plasma PCS in these patients (Supplemental Table 4), it has been shown that plasma metabolites predict the α-diversity of the gut bacterial flora (68), suggesting that whenever PCS concentrations are elevated in the plasma, this would imply that the gut bacterial flora is enriched in bacterial species able to produce PCS and vice versa.

PLWH have gut microbiome flora containing more proinflammatory bacteria than HIV-1–negative individuals (23, 26, 69). It has been suggested that this shift in the microbiome contributes to the increased microbial translocation associated with systemic inflammation and immune activation that characterize all stages of HIV disease (23, 26, 69). The use of antibiotic treatment to deplete microbial flora to prevent eventual bacterial translocation and inflammation was recently explored in the clinical trial ACTG A5286 (70, 71) that includes INR participants. Although the antibiotic rifaximin reduced levels of inflammatory markers (70), it failed to significantly alter microbial diversity within INRs after 4 weeks of treatment (71). Alternative approaches have been used to lower levels of inflammatory markers in PLWH, such as fecal microbiota transplantation (apparently antibiotic treatment is a precondition for implanting fecal transplants) (72, 73), or probiotic intervention that succeeded (74) or failed (75) in lowering the inflammation marker levels. Recently, diet approaches were employed as interventions to lower inflammatory markers and reduce T cell activation in INR patients (76–78). For instance, the Mediterranean diet, with low-protein input, has been shown to improve metabolic parameters, immune activation, Treg function, and increase the α-diversity of the gut microbiota in INRs (78). In CKD patients as well, a low-protein diet decreases PCS plasma levels accompanied with restoration of intestinal permeability (79). It is likely that interventions that increase saccharolytic over proteolytic bacteria is beneficial in lowering inflammation and T cell activation detected in INRs. As a corollary, enrichment of proteolytic gut bacterial flora that metabolize proteins to generate phenols (precursors of *p*-cresol) and indoles (precursors of IS; refs. 80, 81) may have detrimental effects, especially within the elderly HIV-1–infected population, as PCS and IS, among other metabolites mostly derived from amino acid degradation by the commensal flora, are linked to morbid outcome in healthy elderly participants (82). The heightened plasma levels of PCS and IS reported here may contribute to the high risk of comorbidity and mortality often linked to INR patients.

## Methods

### Reagents and flow cytometric assays

Frozen PBMCs and plasma were used in this study. PCS (A8895) and IS (13875) were purchased from ApexBio and Sigma-Aldrich, respectively. For T cell sorting, a Memory CD4^+^ T Cell Isolation Kit (130-091-893) was purchased from Miltenyi Biotec. One to two million frozen sorted cell pellets and 200 μL of plasma were shipped to Metabolon, Inc. for metabolic profiling.

Cell culture proliferation and apoptosis assays were performed as described previously (11, 83). Briefly, 500,000 PBMCs were labeled with CTV (Invitrogen, Thermo Fisher Scientific) and stimulated with anti-CD3/anti-CD28 (T Cell TransAct, 130-111-160, Miltenyi Biotec) for 72 hours, with gradient concentrations of PCS or IS in 2% FBS–containing RPMI media and then collected and stained with anti-CD3–PE (561803, BD), anti-CD4 (560345, BD), and annexin V (556547, BD). Cells were acquired on a BD Fortessa flow cytometer and analyzed by FlowJo software (BD). Statistical analyses were performed using GraphPad Prism software.

A 21-color flow cytometric panel was used to quantitate mitochondrial mass in the different immune cell populations in vitro. Antibodies against T cells were anti-CD3 Pacific Blue (clone UCHT1, catalog 558117, BD), anti-CD4 APC-H7 (clone RPA-T4, catalog 560158, BD), and anti-CD8 BUV496 (clone RPA-T8, catalog 612943, BD). Antibodies against memory T cells were anti-CD45RO BUV395 (clone UCHL1, catalog 564291, BD), anti-CD45RA BUV737 (clone HI100, catalog 612846, BD), anti-CD27 BV605 (clone L128, catalog 562656, BD), anti-CCR7 PE-Cy7 (clone 3D12, catalog 557648, BD), and anti-CD95 PE-Cy5 (clone DX2, catalog 559773, BD). The antibody used for antigen presentation was anti–HLA-DR BV711 (clone G46-6, catalog 563696, BD). Antibodies against myeloid dendritic cells and plasmacytoid dendritic cells were anti-CD11c BUV615 (clone 3.9, catalog 752323, BD) and anti-CD123 PERCy5.5 (clone 6H6, catalog 306016, BioLegend). Antibodies against monocytes were anti-CD14 BV480 (clone M0P9, catalog 566141, BD) and anti-CD16 R718 (clone 3G8, catalog 566970, BD). The antibody against NK cells was anti-CD56 BV786 (clone NCAM16.2, catalog 564058, BD). Antibodies against B cells were anti-CD19 BV570 (clone HIB19, catalog 302236, BioLegend) and anti-CD21 APC (clone B-ly4, catalog 561767, BD). Antibodies against myeloid-derived suppressor cells were anti-CD11b BV650 (clone ICRF44, catalog 740566, BD) and anti-CD15 BUV563 (clone W6D3, catalog 741417, BD). Living cells were identified using a Fixable Blue Dead Cell Stain Kit (catalog L23105, Invitrogen, Thermo Fisher Scientific). Proliferating cells were evaluated by using an antibody against transferrin receptor 1 (CD71) conjugated with BUV805 (clone L01.1, catalog 748308, BD). MitoTracker green was used to evaluate mitochondrial mass (catalog M7514, Invitrogen, Thermo Fisher Scientific). Cells were acquired using the BD FACSymphony flow cytometer and analyzed using FlowJo.

### PCS and IS concentration evaluation in the plasma

Reference standardization (84) was used to quantify PCS and IS in study samples. Reference concentrations of PCS and IS were determined using a method of standards addition and analyzed as previously described (84).

### Measurement of plasma biomarkers

Plasma IL-6 in the CLIF cohort was measured using a high-sensitivity ELISA kit for human IL-6 (Quantikine HS) from R&D Systems. Plasma levels of IP-10 were measured by IP-10 ELISA (Quantikine, R&D Systems). Plasma levels of human IFABP were measured using a DuoSet ELISA Development kit from R&D Systems following the manufacturer’s protocol. For plasma LPS measurement, plasma samples were diluted to 10% or 20% with endotoxin-free water and then heated to 85°C for 15 minutes to denature plasma proteins. They were then quantified using a commercially available Limulus Amebocyte Lysate (LAL) assay (QCL-1000, Lonza) according to the manufacturer’s protocol. Plasma HA was measured using a Biovision HA ELISA kit (Echelon Biosciences Inc).

### EM

Frozen PBMCs from participants were thawed and sorted memory CD45RO^+^CD3^+^CD4^+^ T cells (4 × 10^6^) were fixed by immersion in 2.5% glutaraldehyde in 0.1 M cacodylate buffer (pH 7.4) for 1 hour at room temperature. The cell pellets were then fixed for 2 hours at room temperature by immersion in freshly prepared triple-aldehyde DMSO (85–87). Cell pellets were postfixed in ferrocyanide-reduced osmium tetroxide. Another water rinse was followed by an overnight soak in acidified uranyl acetate. After again rinsing in distilled water, the sample blocks were dehydrated in increasing concentrations of ethanol, passed through propylene oxide, and embedded in EMBed 812 resin (Electron Microscopy Sciences). Thin sections were sequentially stained with acidified uranyl acetate followed by a triple lead stain (88). These sections were examined in a FEI Tecnai Spirit (T12) transmission electron microscope with a Gatan US4000 4k × 4k CCD. A total of 20 EMI sections from 2 independent experiments were analyzed to generate the data displayed in Figure 4.

### Microbiome analyses

Fecal samples were collected from 6 HCs and 12 HIV-1–infected persons receiving ART for 2 or more years: 6 INRs (CD4^+^ T cells <350/μL) and 6 IRs (CD4^+^ T cells >500/μL) (Supplemental Table 4).

#### Amplicon library preparation.

DNA was extracted and sequenced by Ion-Torrent (Life Technologies) using the bacterial *16S* rDNA V4 region. The reactions were carried out on 100 ng of DNA template in 50 μL (final volume) reaction mixtures consisting of DreamTaq Green PCR Master Mix (Thermo Fisher Scientific), 0.1 g/L bovine serum albumin, 1% DMSO, 6 mM MgCl_2_, and a final primer concentration of 400 nM. Initial denaturation at 94°C for 3 minutes was followed by 35 cycles of denaturation for 30 seconds each at 94°C, annealing at 50°C for 30 seconds, and extension at 72°C for 1 minute. Following the 35 cycles, there was a final extension time of 5 minutes at 72°C. The V4 region of the *16S* rRNA gene was amplified using *16S*-515F (GTGCCAGCMGCCGCGGTAA) and *16S*-806R (GGACTACHVGGGTWTCTAAT) primers. The reactions were carried out on 100 ng of template DNA in a 50 μL (final volume) reaction mixture consisting of DreamTaq Green PCR Master Mix, 0.1 g/L bovine serum albumin, 1% DMSO, 6 mM MgCl_2_, and a final primer concentration of 400 nM. Initial denaturation at 94°C for 3 minutes was followed by 30 cycles of denaturation for 30 seconds each at 94°C, annealing at 50°C for 30 seconds, and extension at 72°C for 1 minute. Following the 30 cycles, there was a final extension time of 5 minutes at 72°C. The size and quality of amplicons was screened by 1.5% Tris-acetate EDTA agarose gel electrophoresis, using 100 V for 45 minutes followed by staining with ethidium bromide.

The PCR products were sheared for 20 minutes using an Ion Shear Plus Fragment Library Kit (Life Technologies). The amplicon library was generated with sheared PCR products using Ion Plus Fragment Library kits (<350 bp) according to the manufacturer’s instructions. The library was barcoded with Ion Xpress Barcode Adapters and ligated with the A and P1 adaptors.

#### Sequencing classification and analysis.

The adapted barcoded libraries were equalized using the Ion Library Equalizer kit to a final concentration of 100 pM. Once equalized, the samples were pooled and diluted to 26 pM, and attached to the surface of ion sphere particles (ISPs) using an Ion PGM Template OT2 200bp kit v2 (Life Technologies) according to the manufacturer’s instructions via emulsion PCR. Quality of ISP templates was checked using an Ion Sphere Quality Control Kit (part no. 4468656) with the Qubit 2.0 device. Sequencing of the pooled libraries was carried out on the Ion-Torrent Personal Genome Machine (PGM) system using the Ion Sequencing 200 kit v2 (all from Life Technologies) for 150 cycles (600 flows), with a 318 chip following the manufacturer’s instructions. Demultiplexing and classification were performed using the Qiime 1.6 platform (http://qiime.org/). The resulting sequence data were trimmed to remove adapters, barcodes, and primers during the demultiplexing process. Reads below Q25 Phred score and read length below 100 bp were removed (89). De novo operational taxonomic units (OTUs) were clustered using Uclust algorithm and defined by 97% sequence similarity (90). Classification at the species level was referenced using the UNITE 5.8s database (https://unite.ut.ee/sh_files/UNITE_public_10.09.2014.fasta.zip; accessed April 25, 2014) and taxa assigned using the nBlast method (https://blast.ncbi.nlm.nih.gov/Blast.cgi) with a 90% confidence cutoff.

### Statistics

In the metabolic analysis, levels of each metabolite were normalized to cell lysate protein concentrations and compared among the groups. Out of the 165 metabolites detected, 127 were statistically significantly diminished or enriched among the groups after *P*-value correction with the Benjamini-Hochberg test. For multiple-group statistical analysis, *P* values were generated by the nonparametric Kruskal-Wallis test and adjusted using Dunn’s multiple-comparison test. *P* values were calculated using a 2-tailed Mann-Whitney *t* test for 2 group comparison. All statistical analyses were performed using GraphPad Prism software.

### Study approval

We studied multiple cohorts of IRs and INRs listed in Supplemental Tables 1–4. Cohort 1 are all Russian HIV-1–infected IRs and INRs and relatively young compared with the other cohorts displayed in Supplemental Tables 2, 3, and 4. Participants provided written informed consent following a protocol approved by the Institutional Review Boards and in accordance with the Declaration of Helsinki.

## Author contributions

SAY conceived and planned the experiments, which were performed by BF, ACDS, GPS, EVS, and LBK. SAY wrote the manuscript, which was revised and edited by KVS, EVS, CS, VCM, MAD, and RPS. KVS, EVS, and LBK provided the Russian cohort samples. PPH and SGD provided the SCOPE cohort samples and BR the CLIF cohort samples. BR and CS collected the stool samples. MR and MAG generated the *16S* data. LNR, ADB, and SAY analyzed the *16S* data. AAS, KG, and RPS provided data on PCS, IS, TMAO, and PAG for the SCOPE and CLIF cohorts. KHL and DPJ quantified and analyzed PCS and IS in plasma of the cohorts studied.

## Supplementary Material

Supplemental data

## Version 1 03/22/2022

In-Press Preview

## Version 2 05/02/2022

Electronic publication
