# Supplementary material for: Gut-derived bacterial toxins impair memory CD4+ T cell mitochondrial function in HIV-1 infection
Source: J Clin Invest. 2022 May 2;132(9):e149571. doi: 10.1172/JCI149571 (PMC9057623; doi:10.1172/JCI149571)
Supplement: Supplemental data [file jci-132-149571-s018.pdf]

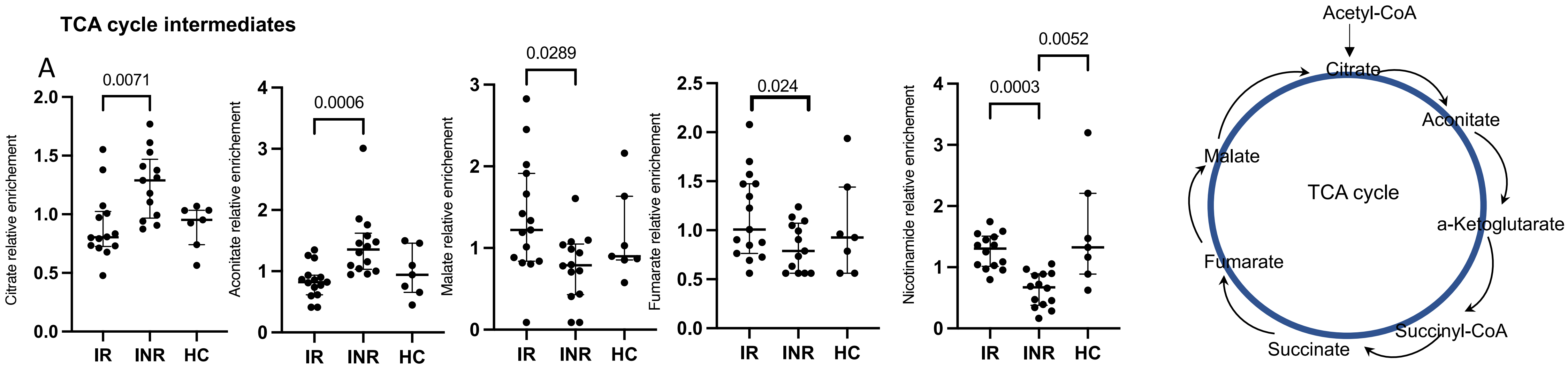

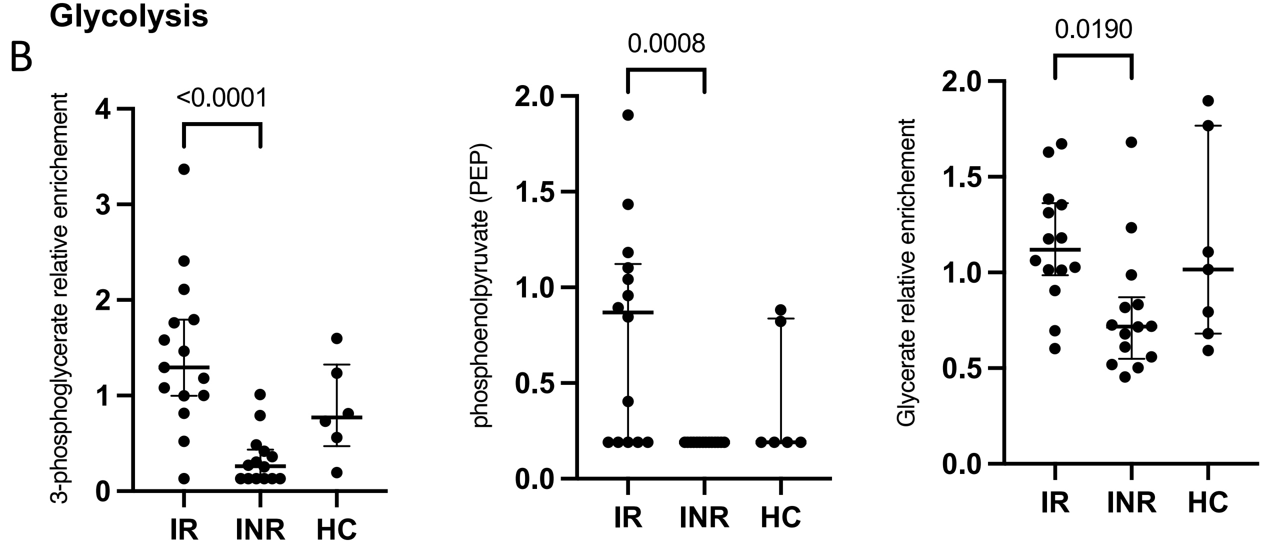

Supplementary Figure 1: Energy production and TCA cycle intermediate quantification by UPLC-MS/MS analysis on lysates from 1 to 2M sorted memory CD4-T cells from IR (n=15), INR (n=14), and HC (n=7). (A) Dot plots with median and Interquartile are shown for TCA cycle intermediates. (B) Dot plots with median and Interquartile intermediates of glycolysis metabolites detected. P values generated by Kruskal-Wallis Dunn's multiple comparisons test.

Amino Acids

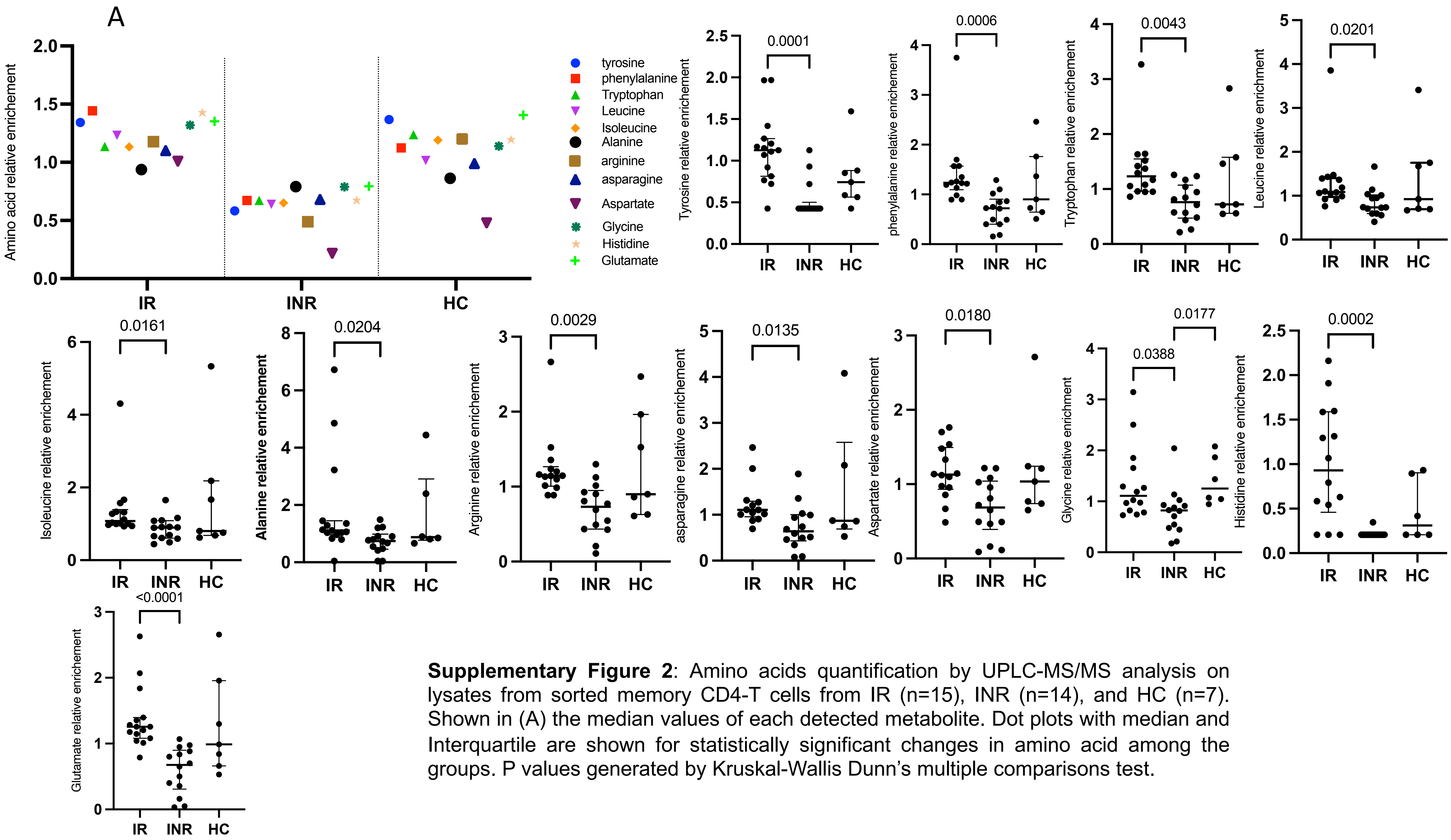

Supplementary Figure 2: Amino acids quantification by UPLC-MS/MS analysis on lysates from sorted memory CD4-T cells from IR (n=15), INR (n=14), and HC (n=7). Shown in (A) the median values of each detected metabolite. Dot plots with median and Interquartile are shown for statistically significant changes in amino acid among the groups. P values generated by Kruskal-Wallis Dunn's multiple comparisons test.

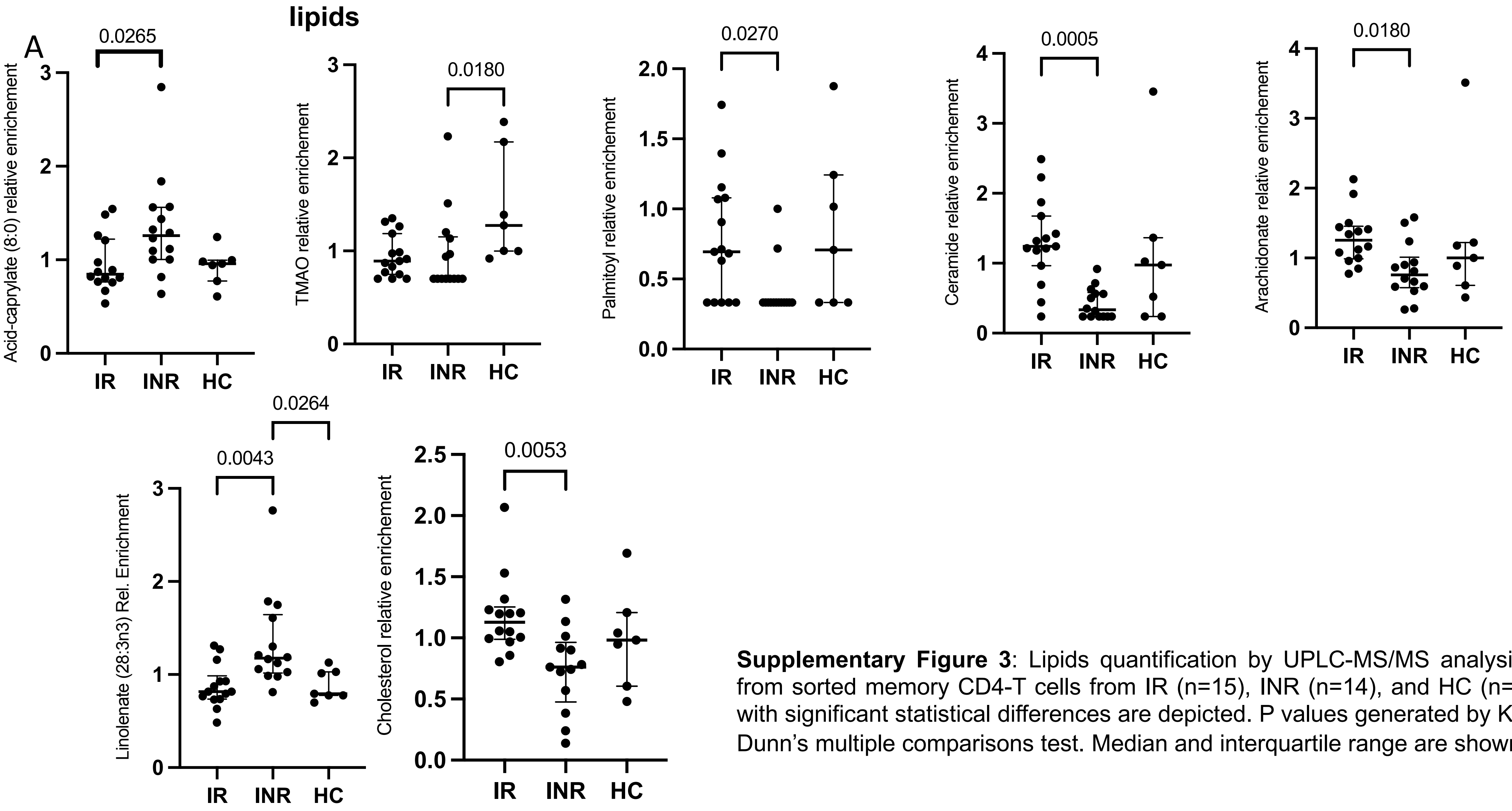

Supplementary Figure 3: Lipids quantification by UPLC-MS/MS analysis on lysates from sorted memory CD4-T cells from IR (n=15), INR (n=14), and HC (n=7). Dot plots with significant statistical differences are depicted. P values generated by Kruskal-Wallis Dunn's multiple comparisons test. Median and interquartile range are shown.

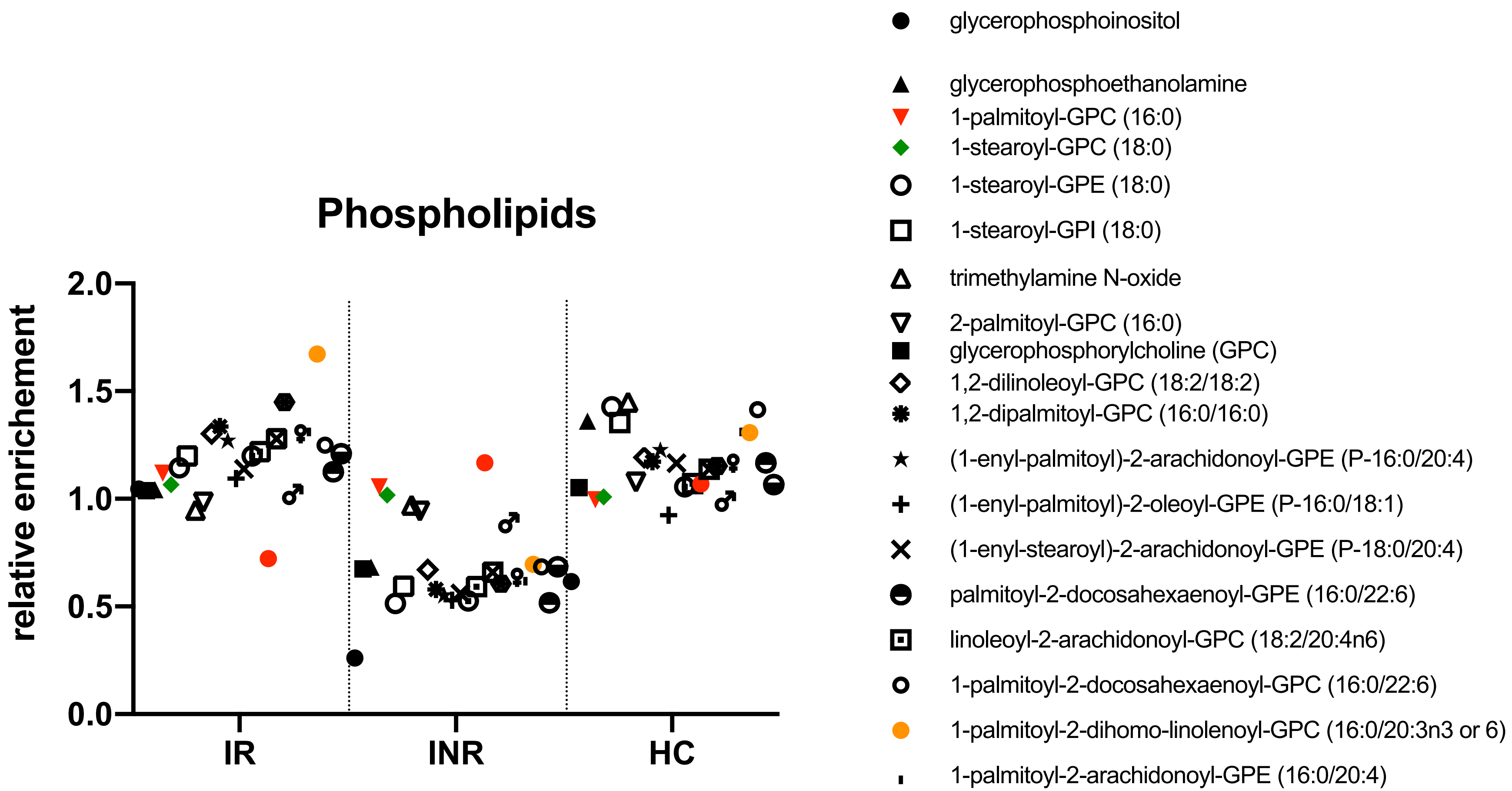

Supplementary Figure 4: phospholipids quantification by UPLC-MS/MS analysis on lysates from sorted memory CD4-T cells from IR (n=15), INR (n=14), and HC (n=7). Shown is the median relative enrichment in each group of IR, INR, and HC. All phospholipids presented here are significantly ($p<0.05$) downregulated or upregulated among the groups. P values generated by Kruskal-Wallis Dunn's multiple comparisons test.

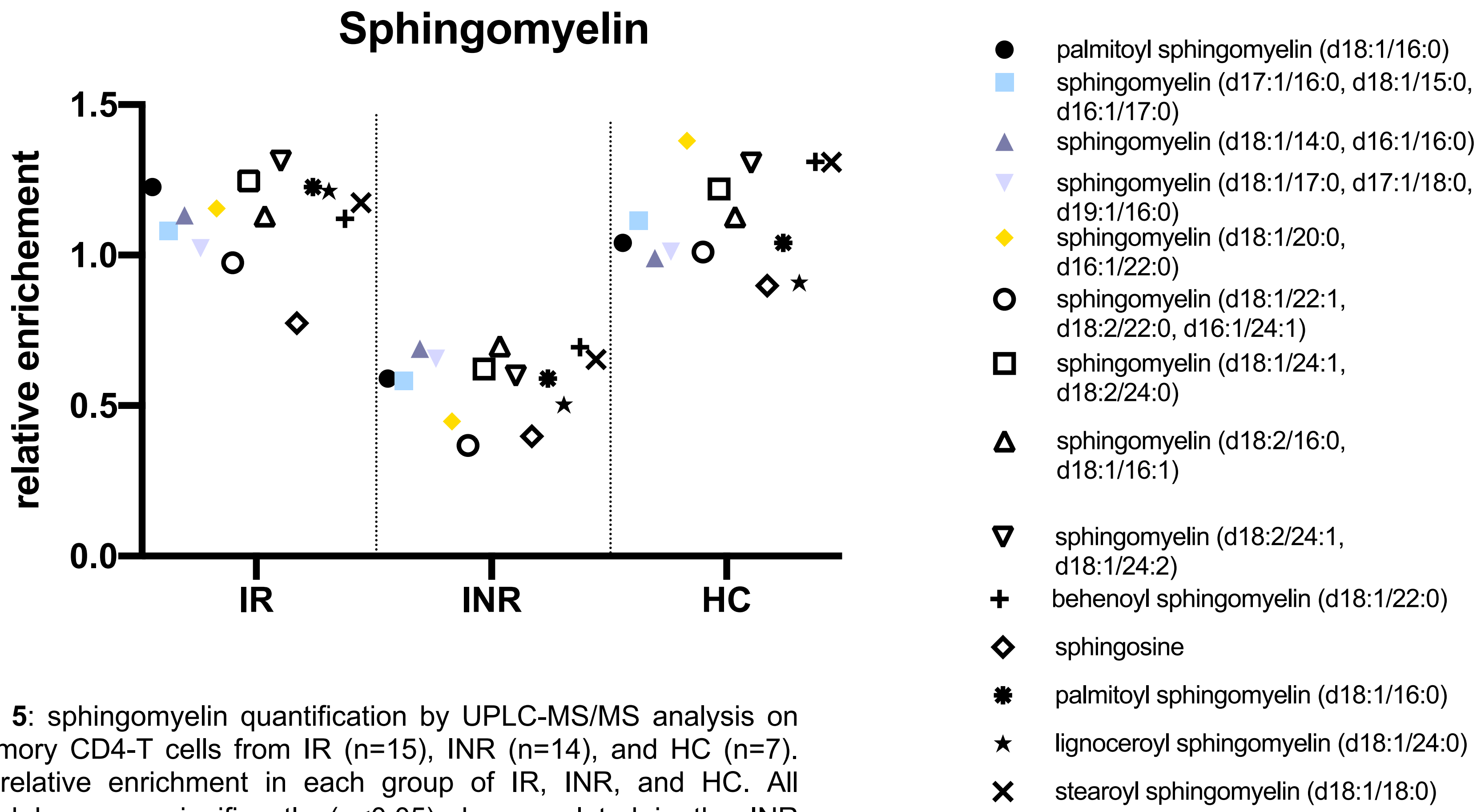

Supplementary Figure 5: sphingomyelin quantification by UPLC-MS/MS analysis on lysates from sorted memory CD4-T cells from IR (n=15), INR (n=14), and HC (n=7). Shown is the median relative enrichment in each group of IR, INR, and HC. All sphingomyelin presented here are significantly ($p<0.05$) downregulated in the INR samples. P values generated by Kruskal-Wallis Dunn's multiple comparisons test.

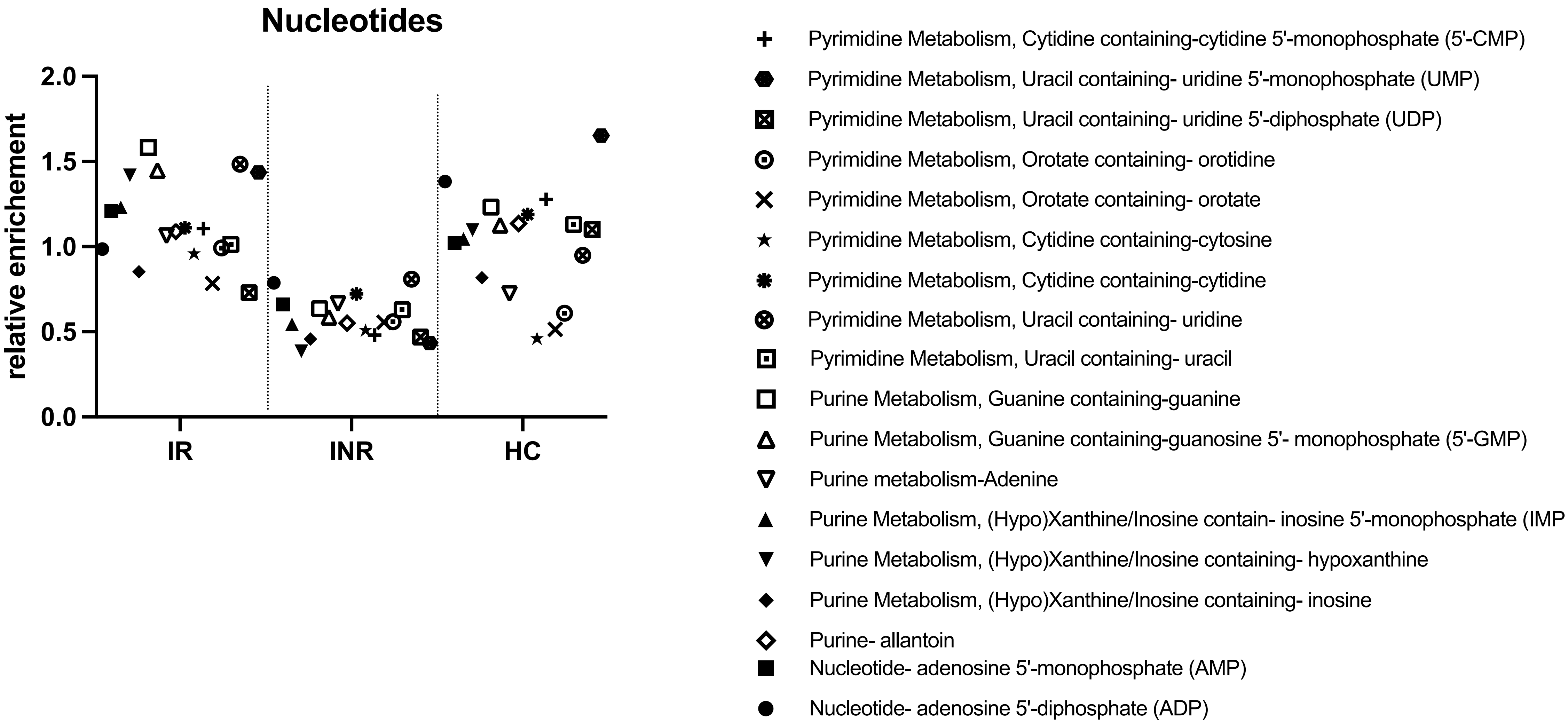

Supplementary Figure 6: Nucleotides quantification by UPLC-MS/MS analysis on lysates from sorted memory CD4-T cells from IR (n=15), INR (n=14), and HC (n=7). Shown is the median relative enrichment in each group of IR, INR, and HC. All pyrimidines and purines presented in here are significantly ($p<0.05$) up or downregulated among the groups. P values generated by Kruskal-Wallis Dunn’s multiple comparisons test.

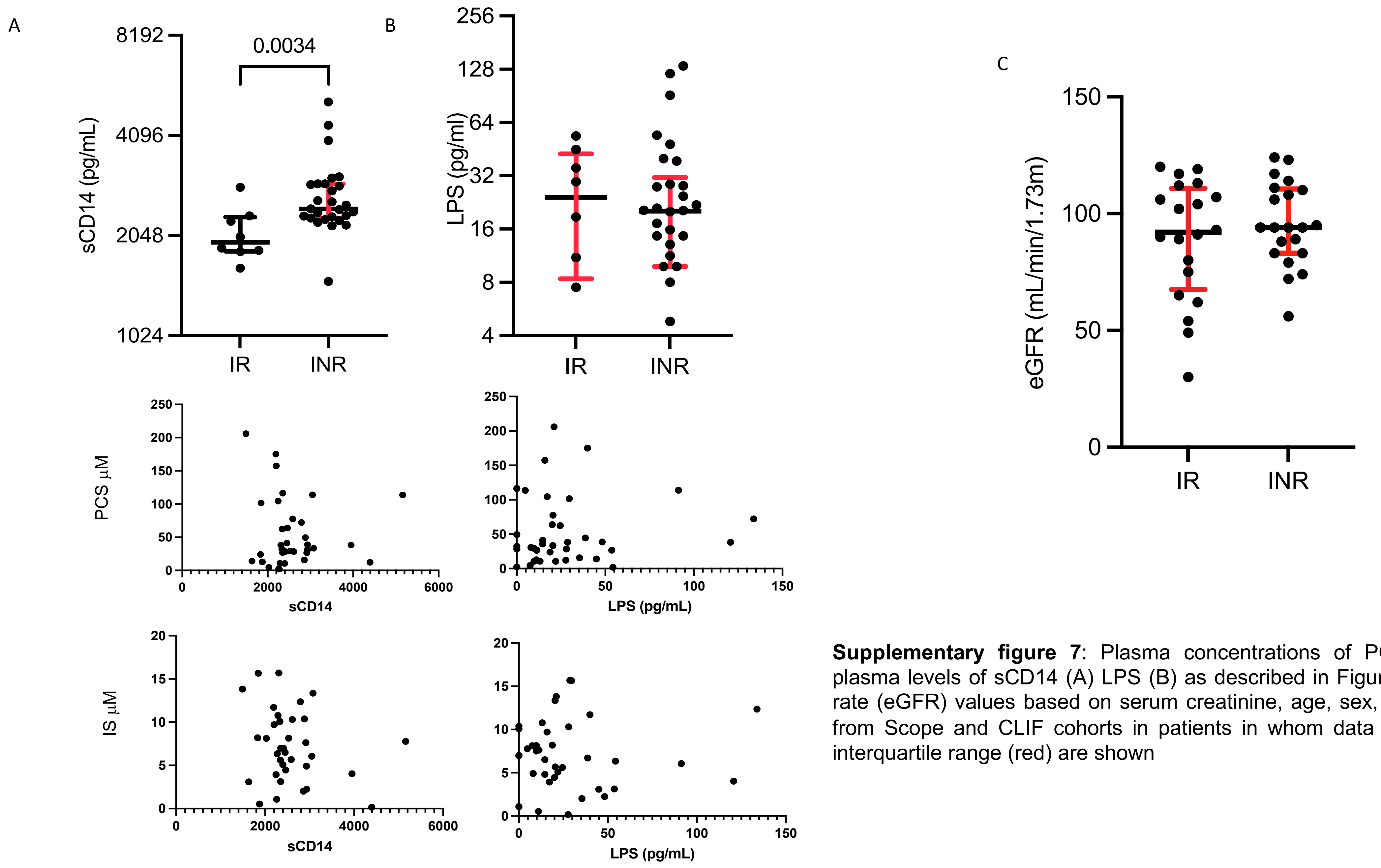

Supplementary figure 7: Plasma concentrations of PCS and IS correlation with plasma levels of sCD14 (A) LPS (B) as described in Figure 2. (C) Glomerular filtration rate (eGFR) values based on serum creatinine, age, sex, and ethnicity, in IR and INR from Scope and CLIF cohorts in patients in whom data were available. Median and interquartile range (red) are shown

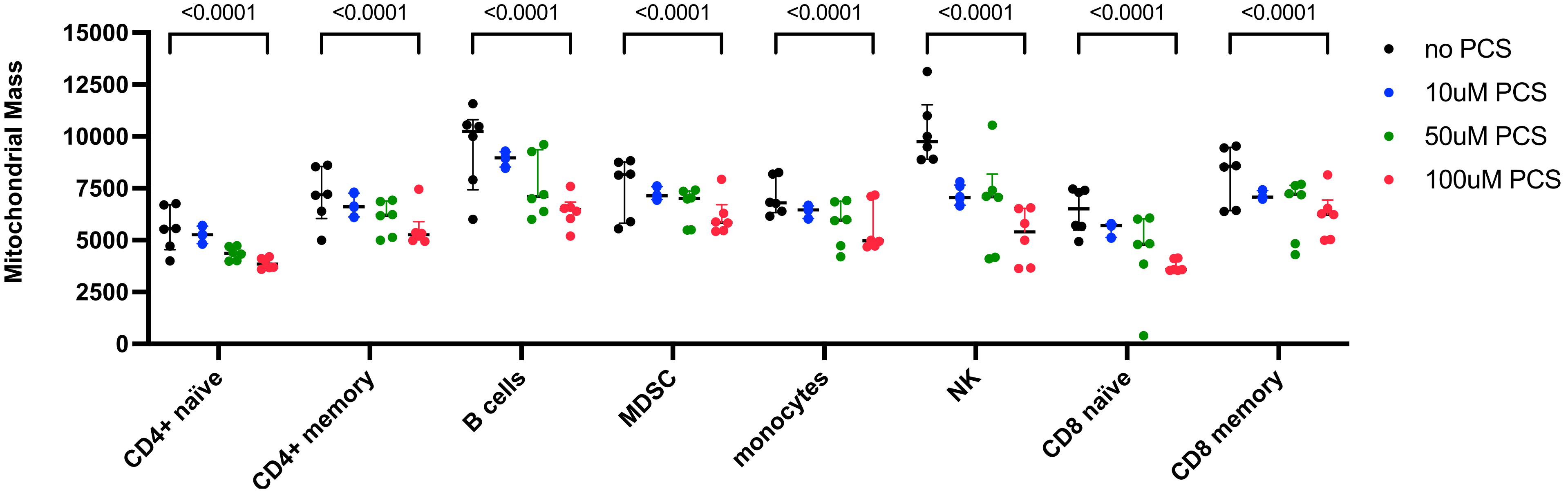

Supplementary figure 8: Effect of PCS on the mitochondrial mass of different subsets of Immune cells. A 21-color flow cytometric assay (see methods) was used to monitor the mitochondrial mass (y-axis) on the different subsets of Immune cells. PBMCs from 6 healthy individuals were treated with gradient concentration of PCS for 72h followed by staining with antibodies and acquisition on BD symphony instrument. P values generated by Kruskal-Wallis multiple comparisons Dunn test. Median and interquartile range are shown.
